# Dietary fennel (*Foeniculum vulgare Mill*) seeds supplementation affects yield, fatty acid composition and flavour profile of milk and cheese in grazing goats

**DOI:** 10.1007/s11250-025-04456-x

**Published:** 2025-05-08

**Authors:** Piera Iommelli, Nadia Musco, Pietro Lombardi, Anna Antonella Spina, Valeria Maria Morittu, Fiorella Sarubbi, Vincenzo Tufarelli, Edmondo Ceci, Federico Infascelli, Raffaella Tudisco

**Affiliations:** 1 Department of Veterinary Medicine and Animal Production, University of Napoli Federico II, 80100 Naples, Italy; 2 Department of Health Science, University “Magna Graecia” of Catanzaro, Catanzaro, Italy; 3 Department of Medical and Surgical Sciences, Magna Græcia University of Catanzaro, Catanzaro, Italy; 4 Institute for the Animal Production System in the Mediterranean Environment, National Research Council, 80055 Portici, Italy; 5 Department of Precision and Regenerative Medicine and Jonian Area, Section of Veterinary Science and Animal Production, University of Bari Aldo Moro, 70010 Valenzano, Italy; 6 Department of Veterinary Medicine, University of Bari Aldo Moro, 70010 Valenzano, Italy

**Keywords:** Goat milk, Fatty acid profile, Fennel seeds, VOCs, Cheese

## Abstract

Fennel (*Foeniculum vulgare Mill*) is an annual plant belonging to the family of *Apiaceae*, widely used in Mediterranean areas for its aromatic and medical properties, especially for carminative, digestive and galactagogue effects. In this trial, 20 multiparous goats homogeneous for body weight (BW: 50.0 ± 2 kg), parity (3rd) and milk yield (1940 ± 120 g/head/day), were randomly allocated into two groups (C: control; F: fennel) fed on a permanent pasture (9:00 am to 4:00 pm). In the pen both groups received 400 g of concentrate mixture (barley and corn meals) and group F diet was supplemented with 15 g/head/day of organic fennel seeds. From the beginning of May until September, milk yield was measured daily, and samples of milk and pasture were collected monthly and analysed, along with concentrate, for their chemical composition and fatty acid profile. Cheese samples were obtained at the beginning and at the end of the trial and analysed for chemical composition, fatty acid and VOCs profile. Milk yield was significantly higher in group F (1809.6 g vs 1418.3 g for group F and C respectively), whereas the solid content did not differ between groups. Milk fatty acid profile differed between groups, especially for the content of MUFA, PUFA, and SFA. Cheese production and composition also was different for yield, fatty acid profile and VOCs composition between the groups. Indeed, the cheese of group F had higher antioxidant capacity and 4 aromatic compounds which were completely absent in the cheese of group C. These results confirm the galactagogue activity of fennel seeds in dairy goats and suggest their potential role as feed additive in grazing system to enhance production in terms of yield and antioxidant activity.

## Introduction

Over the years, the use of medicinal plants in livestock nutrition has been a common practice in many parts of the world, mainly adopted directly by farmers to correct deficiencies in the diet (Shai et al. 2022) or to treat some pathologies with natural remedies (Mayer et al. 2014). Nevertheless, in the last years the use of synthetic chemicals has become prevalent and in response, public awareness of the environmental and health risk has increased the drive to find more natural alternatives (Rochfort et al. 2008). As a matter of fact, recently, the interest in ethnoveterinary medicine has grown leading to more than thousand papers published in European countries between 1990 and 2013 which considered the use of medicinal plants in ruminants (Mayer et al. 2014).

Medicinal plants can be defined as the plants that possess therapeutic properties or exert beneficial pharmacological effects on human or animal body (Namdeo 2018). Among them, the galactagogue group appears to be of particular interest in dairy ruminant since these plants are characterized by different bioactive compounds able to enhance milk production. One of the main representatives of this group is fennel (*Foeniculum vulgare Mill*) which has been traditionally used as galactagogue in women since ancient times. Fennel is still largely used in popular medicine, especially in Mediterranean countries and in Iran (Akbar 2020a); it is indeed one of the most commonly consumed herbs by more than a quarter of Italian pregnant women every day for at least 3 months during pregnancy (Facchinetti et al. 2012).

Fennel (*Foeniculum vulgare Mill*) is an aromatic plant belonging to the family of *Apiaceae* traditionally used as a medicinal plant in human healthcare (Badgujar et al. 2014), employed also for its carminative, digestive and diuretic properties (Rather et al. 2012).

Its main phytocomponents are phenolic compounds, glycosides and other volatile organic compounds (VOCs) among which the trans-anethole, estragole, limonene and fenchone are the most abundant and of particular importance for their medical properties (Rather et al. 2016).

In particular, fennel’s galactagogue effects are related to the great content in trans-anethole, a monoterpene able to inhibit the link of dopamine with its own receptors acting as antagonist (Hassanzadeh et al. 2022) hence reducing the effects of dopamine on prolactin (PRL) (Benker and Jaspers 1990). Through this process, PRL works for a longer time enhancing the production of milk by a direct effect on mammary gland (Lacasse et al. 2012).

Several studies demonstrated that supplementing breastfeeding women diet with fennel seeds increases both milk production and the weight of the newborn (Akbar 2020b; Alachkar et al. 2011; Facchinetti et al. 2012) whereas in animal field the studies are much fewer, especially on dairy ruminants. El-Hendawy et al. (2018) tested fennel seeds as supplement for Damascus goats showing an improvement in reproductive parameters, milk yield (MY) and chemical composition, while Moosavi-Zadeh et al. (2023) investigated fennel effects on dairy cows’ feeding by evaluating milk productive characteristics. Both in cows and in goats, fennel supplementation increased milk yield, while different results were observed for the content of lactose, protein and fat. As far as we know from the literature, few studies have been made on the characteristics of cheese obtained by animals supplemented with fennel seeds, while the effects of adding fennel (as it is or as aqueous extract or as essential oil) during cheesemaking are more investigated and consistent. Previous studies have shown that incorporating antioxidant additives into the diet of animals can enhance the nutritional properties of their food (Esposito et al. 2023; Morittu et al. 2021). The addition of aqueous extracts of fennel during cheesemaking, indeed, improved stability and shelf-life of the products (Mansha Rafiq and Ghosh 2021) by increasing its antioxidant properties (Caleja et al. 2015). Similar results were observed in cheese obtained by fennel supplemented goats (Ismail 2004) where the chemical, microbiological and organoleptic properties of Domiati cheese were evaluated. A significant decrease in lipolysis and proteolytic phenomena as in the viable bacterial count of coliform and spore-forming bacteria was observed. To the best of our knowledge, no studies have been conducted on the influence of fennel seed supplementation on the flavour compounds of goat’s cheese.

Fennel has been chosen for this work for its galactagogue properties with the aim to evaluate its effects on grazing goats’ performances and to contribute to the study of medicinal plants potential in ruminants’ nutrition.

Therefore, the present work is aimed to evaluate the effects of fennel seed supplementation in grazing goats on milk and cheese yield, chemical composition, fatty acid profile and flavour composition.

We also hypothesize that the galactagogue properties of fennel seeds would improve goats’ productive performance over lactation and would affect cheese aromatic profile enhancing its nutritional characteristics.

## Material and methods

### Experimental design

The trial was performed at Azienda Zootecnica Antonio Amato located in Casaletto Spartano (SA, Italy; 832 m a.s.l. (40°09’ N; 15°37’E) on 20 multiparous non-pregnant Murciana goats from April to September 2022, according to the Animal Welfare and Good Clinical Practice (Directive 2010/63/EU) and was approved by the local Animal Ethic Committee (protocol number: PG/2019/0070006).

Sixty days after kidding, the goats, homogeneous for age, date of kidding, body weight (BW = 50 kg ± 2.0 kg) and MY (1940 ± 120 g/day), were randomly allocated into two groups of ten goats each (C: control and F: fennel). All the animals one week after kidding were fed on a permanent pasture from 9.00 to 16.00 and received 400 g/head/day of corn and barley meals (50/50) and 2.2% as concentrate fed of vitamin-mineral mix in individual pen; in addition, group F received 15 g/head/day of grinded organic dry fennel seeds (*Foeniculum vulgare Mill*) which was mixed into the concentrate before being offered to the animals. All the animals had free access to fresh water and feed refusal were daily measured. After 10 days of adaptation period to the diets the trial has started. The diet of all the grazing goats were formulated to satisfy their energy requirements according to Tudisco et al. (2014) as follows: energy requirements for maintenance 0.0365 UFL/kg metabolic weight (MW:BW^0.75^); energy requirements for milk production 0.41 UFL/kg fat-corrected milk (FCM, 4% fat). Therefore, total energy requirement was 1.25 UFL (0.68 UFL maintenance + 0.57 UFL milk production) and since the average pasture DM intake in the inlands of South Italy is 20 g/kg BW (Iommelli et al. 2022), the goats, which weighed 50 kg, ingested around 1 kg of pasture, corresponding to an average of 0,78 UFL and the deficit of 0.4 UFL was met by the concentrate. Fennel seeds were characterized for antioxidant activity (total phenolic content (TPC), 2,2 -azino-bis (3-ethylbenzothiazoline-6-sulfonic acid) (ABTS), and 2,2-diphenyl-1-picrylhydrazyl (DPPH) and VOCs as presented in Table 1.

**Table 1:** Fennel seeds antioxidant activity and volatile organic compounds (VOCs)

VOCs	Fennel seeds
Butanoic acid	0.1
Hexanoic acid	0.1
Nonanoic acid	0.1
Decanoic acid	0.1
Dodecanoic acid	0.1
Tetradecanoic acid	0.1
2,3-Butanediol	0.1
1-butanol-3-methyl	0.1
Phenylethyl alcohol	0.1
Ethyl hexanoate	0.8
Ethyl octanoate	0.4
Ethyl decanoate	0.1
2-Heptanone	0.6
2-Nonanone	0.1
Limonene	14.5
Fenchone	2.5
Estragole	4.3
trans-Anethole	75.5
2-Butanol	0.2
Acetic acid	0.1
Antioxidant activity	
TPC, mg GAE/g	4.06
ABTS, mg/g TE	6.74
DPPH, EC50 µg/ml	12.04

*VOCs* percentage of total volatile organic compounds detected, *TPC* Total phenolic content, *ABTS* 2,20-azino-bis(3-ethylbenzothiazoline-60-sulfonic acid) diammonium salt, *DPPH* 2,2-diphenyl-1-picrylhydrazyl

TPC in samples was determined by the Folin-Ciocalteu colorimetric method described by Taga et al. (1984). The concentration was calculated using gallic acid as standard (Sigma-Aldrich), and the results were expressed as mg gallic acid equivalents (GAE)/g.

To evaluate the antioxidant activity of the samples, the ABTS method was applied according to the procedure described by Re et al. (1999), with minor modifications. In particular, a 7 mM solution of ABTS in 2.45 mM aqueous potassium persulfate was prepared, and after 16 h of incubation in the dark at room temperature, the solution was diluted with ethanol to obtain an absorbance of 1.000 ± 0.020 at 734 nm. Then, 100 µL of the extract, obtained according to Wojdyło et al. (2007), was added to 1000 µL of this solution. Measurements of absorbance were taken at 734 nm after 2.5 min of incubation. In addition, Trolox was used as the reference and activities were expressed as Trolox equivalents (mg/g TE).

The antiradical activity of seeds was measured using DPPH method (Sanchez-Moreno et al. 1998) and expressed as half maximal effective concentration (EC50 (µg/ml)), the concentration necessary for 50% reduction of DPPH.

The VOCs extraction from samples of fennel seeds was performed by solid-phase microextraction (SPME), whereas the GC–MS analysis was carried out with a gas chromatograph (Perkin Elmer, Waltham, MA, USA) coupled with a mass spectrometer (SQ8S; Perkin Elmer, USA). The gas chromatograph was equipped with an Elite-5MS column (Perkin Elmer, USA). A total of 5 g of fennel seeds were mixed with 10 ml of saturated sodium chloride solution (360 g/l), and then 10 μl of internal standard solution (4-methyl-2-heptanone; 10 mg/kg in ethanol) was added. The vials were sealed with a polytetrafluoroethylene-silicone septum (Supelco, Bellefonte, PA, USA) and stirred at 60 °C; VOCs were extracted from the headspace with a divinylbenzene-carboxen-polydimethylsiloxane SPME fiber (Supelco, USA) with an exposition time of 60 min. After adsorption time, the extracted VOCs were thermally desorbed into the gas chromatograph injector splitless mode for 1 min at 250 °C. The oven temperature was held at 50 °C for 1 min, increased at a rate of 3 °C/min up to 200 °C and held for 1 min, and then increased from 200 °C to 250 °C at 100.15 °C/min and held for 15 min. Helium was used as a carrier gas at a flow rate of 1 ml/min. The mass spectrometer operated in electronic impact ionization mode at 70 eV, and data were collected in full scan mode. Source and interface temperature were held at 250 °C. Compounds were identified by comparing their mass spectra with those contained in the National Institute of Standards and Technology (NIST) 14 library (Gaithersburg, MD, USA). The compounds were considered as correctly identified when their spectra presented a library match factor > 85. The VOCs were expressed on percentage of total volatile compounds detected.

### Feeds samples and analysis

Pasture samples were collected weekly as described in Musco et al. (2022) briefly, every week pasture samples (1 kg each) were collected from four different areas of 2.5 m^2^ each, cutting the plants at 3 cm from the ground and then air oven dried at 65 °C. The representative weekly samples were weighted and pooled to obtain monthly samples which were trimmed at 1 mm. Concentrate and fennel seeds were collected on a monthly basis. All feed samples (pasture, concentrate and fennel seeds), were analysed for chemical composition, according to AOAC (2012) procedures, and fibre content as suggested by Van Soest et al. (1991). Feed nutritional value was calculated according to INRA (2018).

Lipid fraction of fennel seeds and pasture was extracted from 15 g of sample using a mixture of chloroform and methanol (2/1 vol/vol) according to Gray et al. (1967). Lipid extract was methylated adding 1 mL of hexane and 0.05 ml of 2 N methanolic KOH. Separation of the methyl esters in forage samples was performed according to Di Trana et al. (2004). Fatty acid methyl esters (FAME) were identified with reference to the retention time of FA standard mixture of Supelco 37 Component FAME Mix (Supelco, Bellafonte, PA). The content of FA was quantified using internal standards (Supelco, Bellafonte, PA) added during the methylation step. Briefly, 100 mg of lipid extract was mixed with 50 µl of 2 N methanolic KOH and 1 ml of hexane containing the internal standards (20 mg/mL) according to Giorgio et al. (2019). Fatty acid methyl esters were fractionated over a CP-SIL883 column (100 × 0.25 mm i.d., film thickness 0.20 μm fused silica; Varian, Palo Alto, CA, USA) in a Shimadzu (Model 2GC17 A) gas chromatograph with a Hewlett-Packard HP 6890 gas system and using flame ionization detection. Helium was used as carrier gas at a constant flow rate of 1.7 ml/min. The oven temperature was programmed as follows: 175 °C, held for 4 min; 175–250 °C at 3 °C/min; and then maintained for 20 min. The injector port and detector temperature were 250 °C. Samples (1 μl) were injected with an auto-sampler. Output signals were identified and quantified from the retention times and peak areas of known calibration standards. Composition was expressed as percentages of the total FA. All determinations were carried out in triplicate.

### Milks samples and analysis

The goats were milked twice a day (at 07:00 a.m. and at 05:00 p.m.) in the milking room by using a portable milking trolley. Starting from 60 days after kidding, MY was daily registered and individual milk samples were collected monthly by proportionally mixing the milk from the two daily milkings.

One-hundred-fifty ml of milk were collected in sterilized falcon tubes and kept at 4 °C until they arrived at the laboratory. Samples (100 ml of milk) were analysed for fat, lactose and protein by Milko Scan 133B (Foss Matic, Hillerod, Denmark) standardized for goat milk. The fatty acid (FA) profile (50 ml of milk) has been determined as described by Tudisco et al. (2014). Briefly, milk fat was extracted by using hexane and isopropane (3/2 v/v) (Hara and Radin 1978). The FAME were prepared by direct transesterification with sulfuric acid and methanol (1:9, v/v) of the lipids (Christie 1993) and then analysed in a gas chromatograph (Agilent technologies, model 5890) fitted with an SP-2560 fused silica capillary column (100 m × 0.25 mm i.d. × 0.2 µm film thickness, Supelco, Inc., Bellefonte, PA, USA). Helium was used as carrier gas and set at a constant pressure of 180 kPa, splitting flow of 50 mL/min, and injection volume of 1 µL. The column parameters were: initial temperature of the column maintained at 170 ◦C for 15 min; then, with an increase of 5 ◦C/min, it was brought up to 240 ◦C. The total execution time was 64 min. Fatty acid peaks were identified using pure methyl ester external standards (Larodan Fine Chemicals, AB, Limhamnsgardens Malmo, Sweden). Additional standards for CLA isomers were obtained from Larodan. Chromatogram peak areas were acquired and calculated by Chemstation software (Agilent,technologies) and expressed as g/100 g, considering 100 g as the summation of the areas of all FAME identified. All determinations were carried out in triplicate.

From the identified FA, atherogenicity (AI) and thrombogenicity (TI) indices were determined, by using Ulbricht and.

Southgate equations (Ulbricht and Southgate 1991). Nutritional indices were calculated as shown in the following equations:

$$AI= (C12:0 + 4 \times C14:0 + C16:0) / (\Sigma n6 PUFA + \Sigma n3 PUFA + MUFA),$$

$$TI = (C14:0 + C16:0 + C18:0) / [(0.5 \times MUFA) + (0.5 \times \Sigma n6 PUFA) + (3 \times \Sigma n3 PUFA) + (\Sigma n3 PUFA/\Sigma n6 PUFA)]$$

### Cheese samples and analysis

Cheeses were sampled after 1 month of treatment and at the end of the trial. They were obtained separately with the milk of the two groups, made from raw milk heated at 37 °C with the addition of rennet. The curd was broken and then reassembled in the ‘fuscelle’, typical wicker baskets which allow the whey to drain for 24 h. Then it was salted (around 2 g of NaCl/100 g of curd) and ripened for 20 days at 10 °C. Four small cheeses (around 500 g) for each sampling period were collected for each group and stored in vacuum packages at −20 °C until analysis.

Cheese yield was calculated dividing the sum of the weight of all the cheeses obtained from the milk of one group by the initial weight of milk.

Cheese samples were thawed over night at 4 °C and then they were grinded homogenized in a stainless-steel blender. The moisture content was determined using oven drying, while the fat and protein contents were analysed using the Gerber and Kjeldahl methods, respectively (AOAC 2002).

Cheese samples FA composition was determined by gas chromatography (GC), according to Bligh and Dyer method and International Organization for Standardization (ISO) as has been reported previously by Papaloukas et al. (2016).

Briefly, aliquots (3 g) of grinded cheese were added with 4,50 ml of chloroform and 9,0 ml of methanol and 3,6 ml of deionized water. This mixture was stirred for 1 min. Subsequently, 4,5 ml of chloroform and of deionized water were added. The tube was shaken for 1 min and then centrifuged for 10 min at 2500 rpm at 4 °C, and the upper organic layer was transferred to a glass test tube. The lower aqueous layer was extracted twice more, with 1,5 mL of n-hexane each time after suspension centrifugation. The pooled hexane layer was evaporated under a stream of nitrogen. The previously dried solution was derivatized according to Moltò-Puigmartì et al. (2007).

The extracted fat was stored at − 20 °C until further analysis. One microliter of each sample was injected into a Thermo GC 6890 gas chromatography system equipped with a flame-ionization detector (Thermo Fisher Scientific., Waltham, Massachusetts, US). FAME from all samples were separated using a 100-m length, 0.25-mm i.d., 0.25-µm capillary column (CP-Sil 88; Chrompack, Middelburg, the Netherlands).

Individual FAME were identified, based both on retention time and on the comparison with a standard mixture of 37 pure components (Supelco 37 Component FAME Mix, Merck Life Science, Milano, S.r.l, Italy) and literature data. The injector temperature was kept at 250 °C and the detector temperature was kept at 250 °C, with an H2 flow of 40 mL/min, air flow of 400 mL/min, and a constant He flow of 45 mL/min. The initial oven temperature was held at 80 °C for 1 min, increased at 5 °C/min to 100 °C, held for 2 min, increased at 10 °C/min to 175 °C, held for 40 min, and then finally increased at 5 °C/min to a final temperature of 240 °C and held for 55 min. Helium was used as the carrier gas at a flow rate of 0,70 ml/min.

The identification of the isomers of the conjugated linoleic acid (CLA) was accomplished by comparing the retention time of each chromatographic peak and those of a mixture of chromatographic standards (Conjugated (9Z,11E)-Linoleic acid; Conjugated (10E,12Z)-Linoleic acid solution, Merck Life Science, Milano, S.r.l, Italy). The internal standard used was the Nonadecanoic Acid Methyl Ester (Merck Life Science, Milano, S.r.l, Italy). The concentrations of FA were expressed as mg/100 mg total FA. All determinations were carried out in triplicate.

The VOCs extraction from samples of cheese was performed with the same method used for analysing fennel seed VOCs content. Total antioxidant capacity (TAC) of cheese extract samples was assessed using the ferric ion reducing antioxidant power assay as following the methods reported Benzie and Strain (1997). The standard curve was constructed using iron sulphate heptahydrate (FeSO_4_·7H_2_O) and data were expressed as mol FeSO_4_ equivalents per kg of cheese.

### Statistical analysis

Data from pasture were analysed by the one-way ANOVA with SPSS IBM software (V29.0.1.0) using as factor the period of sampling; the means were compared using Tukey’s test. Differences were considered statistically significant at *P* < 0.05.

Milk and cheese data were analysed using the two-way ANOVA for repeated measures on SPSS IMB software (V29.0.1.0). In the statistical model, group (G), period (P) and their interaction (G x P) were considered as fixed effects.

Figures were obtained with SPSS IMB software (V29.0.1.0).

## Results

### Diet composition

Feeds chemical composition (g/kg of dry matter (DM)) and nutritive value are reported in Table 2. Pasture had the highest content of protein and fat in July and the highest NDF and the lowest ADF and ADL content in May. The fennel seeds had high fat and crude fibre contents. The ash content of pasture was significantly higher during the sampling of September compared to the other months.

**Table 2:** Feed chemical composition (g/kg of DM) and nutritive value (UFL/kg di DM)

	Pasture						Corn and barley meal	Fennel seeds
Item	May	June	July	August	September	SEM		
CP	112.8 ± 1.6^E^	147.1 ± 0.5^D^	201.0 ± 2.0^A^	169.1 ± 0.3^C^	176.4 ± 2.2^B^	0.79	100.4 ± 2.91	120.9 ± 0.7
Crude fat	21.3 ± 0.3^C^	22.2 ± 0.3^B^	25.6 ± 0.5^A^	22.6 ± 0.4^A^	19.5 ± 0.5^D^	0.05	31.6 ± 0.16	82.4 ± 0.14
CF	26.97 ± 0.19^C^	30.04 ± 0.28^B^	31.43 ± 0.26^A^	31.71 ± 0.30 ^A^	29.80 ± 0.77^B^	0.45	47 ± 2.22	112.3 ± 1.7
Ash	76.4 ± 4.7 ^B^	65.0 ± 1.3 ^B^	61.2 ± 1.0 ^B^	41.4 ± 1.2 ^C^	123.1 ± 3.6^A^	0.73	10.5 ± 0.13	14.8 ± 0.5
NDF	562.0 ± 1.5^A^	481.8 ± 3.6^B^	490.1 ± 5.2^B^	410.6 ± 6.9^C^	475.0 ± 9.1^C^	1.29	/	/
ADF	318.7 ± 2.0^E^	422.0 ± 2.4^D^	453.8 ± 2.3^C^	476.4 ± 1.8^A^	468.3 ± 3.8^B^	1.54	/	/
ADL	98.3 ± 5.3^D^	176.7 ± 5.2^B^	152.5 ± 2.6^C^	197.7 ± 3.7^C^	181.6 ± 11.6^A^	0.93	/	/
UFL	0.77	0.78	0.80	0.77	0.78	0.04	1.06	0.8

*CP* crude protein, *CF* crude fiber, *NDF* neutral detergent fiber, *ADF* acid detergent fiber, *ADL* acid detergent lignin, *UFL* feed unit for lactation, *SEM*: standard error of the mean.

Values within a row with different superscripts differ (*P* < 0.01)

Feeds fatty acid profiles are reported in Table 3. Pasture samples were rich in polyunsaturated fatty acids (PUFA) and in particular in C18:3n3 (alpha-linolenic acid), with the highest values in May and September. On the contrary, saturated fatty acids (SFA) were the lowest at the beginning and at the end of the experimental period. Fennels seeds had low values of SFA and extremely high content of monounsaturated fatty acids (MUFA), mainly composed of C18:1n12 (petroselinic acid) and very low content of alpha-linolenic acid.

**Table 3:** Fatty acid profile (expressed as % on total FA) of fennel seeds and pasture

	Fennel	Pasture					
Item		May	June	July	August	September	SEM
C11:0	6.151	nd	nd	nd	nd	nd	-
C12:0	0.018	0.253^A^	0.226^B^	0.099^D^	0.118^C^	0.079^E^	0.03
C14:0	0.046	0.589^A^	nd	0.499^B^	0.331^D^	0.360^C^	0.51
C16:0	4.183	17.657^D^	19.723^C^	21.567^A^	20.777^B^	16.458^E^	0.19
C18:0	1.399	6.760^A^	5.887^B^	5.217^C^	4.92^E^	4.954^D^	0.20
C22:0	0.018	0.761^D^	2.423^A^	1.638^B^	0.923^C^	0.261^E^	0.67
SFA	12.925	27.378^D^	30.217^B^	30.413^A^	28.617^C^	23.516^E^	0.08
C16:1 trans	0.201	0.567^B^	0.892^A^	0.324^C^	0.136^E^	0.148^D^	0.03
C16:1cis	0.197	0.658^A^	0.494^C^	0.468^D^	0.574^B^	0.373^E^	0.34
C18:1n9	0.33	2.279^E^	3.991^D^	5.760^A^	4.905^C^	5.480^B^	0.04
C18:1n12	75.572	nd	nd	nd	nd	nd	-
C20:1n9	0.146	0.463^C^	0.616^B^	0.373^D^	0.675^A^	0.256^E^	0.32
MUFA	76.416	3.967^E^	6.250^D^	7.398^A^	7.045^B^	6.407^C^	0.35
C18:2n6	9.989	12.495^B^	14.815^A^	11.443^D^	11.233^E^	11.659^C^	0.95
C18:3n3	0.230	54.435^B^	46.556^E^	47.602^D^	51.753^C^	55.360^A^	0.01
C20:3n3	0.200	0.645^A^	0.626^B^	0.524^C^	0.436^D^	0.315^E^	0.03
C20:5n3	0.061	0.522^A^	0.489^B^	0.420^C^	0.418^C^	0.372^D^	0.01
C22:6n3	0.023	0.184^B^	nd	0.124^C^	0.083^D^	1.516^A^	0.18
PUFA	10.658	68.656^B^	63.537^C^	62.190^D^	64.338^C^	70.078^A^	0.82

*SFA* saturated fatty acids, *MUFA* monounsaturated fatty acids, *PUFA* polyunsaturated fatty acids, *SEM* standard error of the means. *Nd* not detected, Values within a row with different superscripts differ (*P* < 0.01)

### Milk yield and chemical composition

**Fig. 1:**
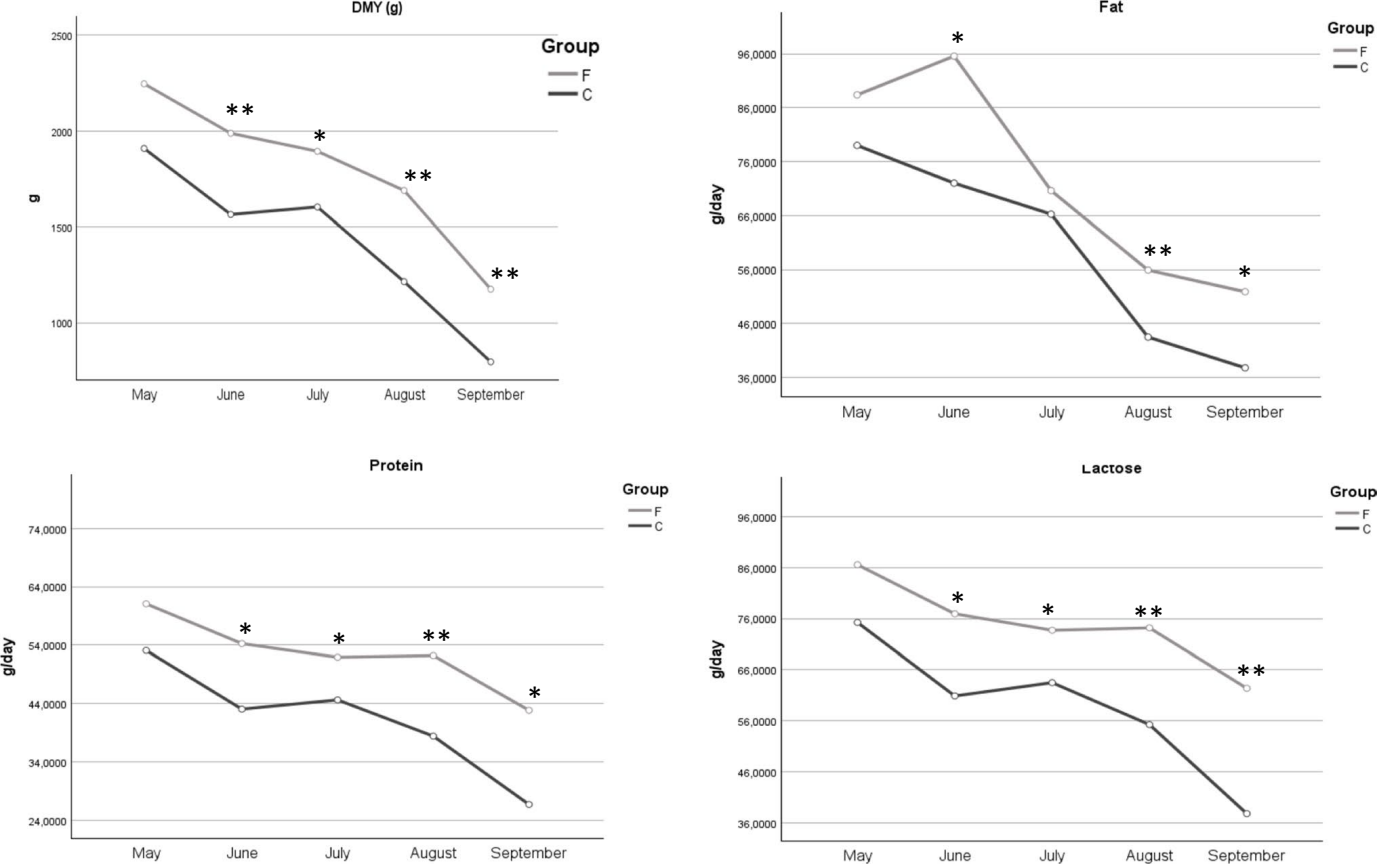
Daily milk yield (DMY), milk lactose, protein and fat yields (g/day) during the trial in the experimental groups * = *P* < 0.05; ** = *P* < 0.01

Goats BW did not change between the groups (Table 4) and no concentrate refusals were detected. MY was significantly higher in group F than in group C (*P* < 0.01) with an increase of 27.5%, whereas no modifications have been detected in fat, lactose and protein percentage (Table 4). Nevertheless, the yields of milk fat, lactose and protein were significantly higher in Fennel group with increases of 21%, 27.5% and 27.1% respectively. The sampling effect was significant for all chemical characteristics but for fat content and no interaction effect was observed. As demonstrated in Fig. 1, a decline in MY was observed in both groups as lactation progressed. The milk solid content, expressed in g/day, exhibited a decline during the lactation period in accordance with the decrease in MY. However, the yields in group F remained higher throughout the entire experimental period.

**Table 4:** Body weight (kg), milk yield (g/day) and chemical composition (% and g/day)

Item	Group C	Group F	Group effect	Period effect	GxP	SEM
			*P -value*			
BW (kg)	51.5	50.2	0.89	0.07	0.98	1.3
MY (g/day)	1418.27	1809.59	< 0.01	< 0.01	0.90	52.84
Fat %	4.31	4.05	0.62	0.88	0.19	0.09
Lactose %	4.24	4.22	0.76	< 0.01	0.39	0.06
Protein %	2.99	2.95	0.62	< 0.01	0.98	0.04
Fat g/d	60.04	72.82	< 0.01	< 0.01	0.52	2.49
Lactose g/d	58.61	74.75	< 0.01	< 0.01	0.53	1.93
Protein g/d	41.26	52.48	< 0.01	< 0.01	0.62	1.37

*BW* body weight, *MY* milk yield, *SEM* standard error of the means

Milk fatty acid profile of the two groups is reported in Table 5. Among the de novo synthesized FA (C4:0 to C15:0), butyric (C4:0), caproic (C6:0), caprylic (C8:0), myristic (C14:0) and mirystoleic (C14:1) were different (*P* < 0.05) between the groups.

**Table 5:** Milk FA profile, classes and ratios expressed as g/100 g of milk fat

Item	Group C	Group F	Group effect	Period effect	G x P	SEM
			*P-value*			
C4:0	3.020	2.461	< 0.01	0.39	0.14	0.0979
C6:0	1.785	1.870	0.035	0.02	0.39	0.0307
C8:0	2.648	2.788	0.028	< 0.01	0.30	0.0414
C10:0	10.393	10.664	0.143	< 0.01	0.45	0.111
C12:0	3.630	3.771	0.188	< 0.01	0.87	0.0589
C14:0	9.191	10.044	< 0.01	0.17	0.22	0.0932
C14:1	0.255	0.207	< 0.01	< 0.01	< 0.01	0.0068
C16:0	28.896	29.540	0.047	0.49	0.81	0.1659
C16:1	0.441	0.383	< 0.01	< 0.01	0.61	0.0157
C18:0	12.407	12.319	0.760	< 0.01	0.17	0.1616
C18:1c6	0.099	0.058	< 0.01	< 0.01	0.02	0.0071
C18:1n9	18.068	17.777	0.082	< 0.01	< 0.01	0.149
C18:1 t9	0.373	0.252	< 0.01	< 0.01	< 0.01	0.0195
C18:1 t11	1.491	1.326	< 0.01	0.49	0.85	0.0267
C18:1c10	0.338	0.301	< 0.01	0.06	0.82	0.0111
C18:1c11	0.179	0.172	0.27	< 0.01	0.53	0.0035
C18:1n12	0.114	0.092	< 0.01	0.25	0.73	0.0032
C18:2 t6	0.273	0.250	0.037	< 0.01	0.14	0.0091
C18:2n6	1.615	1.496	< 0.01	< 0.01	0.16	0.0267
C18:2c9 t11	0.380	0.326	< 0.01	< 0.01	0.45	0.0138
C18:2 t10c12	0.220	0.196	< 0.01	0.01	0.46	0.0039
C18:3n6	0.049	0.045	0.031	0.06	0.10	0.0016
C18:3n3	0.942	0.832	< 0.01	< 0.01	0.36	0.0164
C20:3n6	0.012	0.007	0.03	< 0.01	< 0.01	0.0011
C20:3n3	0.005	0.005	0.40	< 0.01	0.07	0.0002
C20:4	0.138	0.113	< 0.01	0.48	0.70	0.0025
C20:5n3	0.053	0.048	0.04	0.09	0.33	0.00
C22:0	0.133	0.115	0.02	< 0.01	0.28	0.0033
C22:2n6	0.012	0.012	0.21	0.39	0.18	0.0
C22:6n3	0.025	0.026	0.32	0.06	0.021	0.001
C24:1	0.023	0.018	0.73	0.37	0.76	0.00
De novo FA	32.01	32.71	< 0.01	0.02	0.39	0.23
Mixed FA	29.33	29.92	0.18	0.95	0.78	0.16
Preformed FA	38.19	36.73	0.01	0.00	0.38	0.22
SFA	74.10	75.28	0.02	< 0.01	< 0.01	0.15
MUFA	21.59	20.66	0.01	< 0.01	< 0.01	0.15
PUFA	3.79	3.42	0.01	< 0.01	0.07	0.03
n-6	2.14	1.96	< 0.01	< 0.01	0.05	0.02
n-3	1.05	0.93	< 0.01	< 0.01	0.38	0.01
Total CLA	0.60	0.52	< 0.01	< 0.01	0.38	0.01
PUFA/SFA	0.05	0.04	0.17	0.08	0.21	0.05
n-6/n-3	2.06	2.14	0.09	0.19	0.72	0.02
LA/ALA	1.73	1.84	0.01	< 0.01	0.96	0.03
AA/EPA	2.55	2.43	0.01	0.26	0.94	0.05
AI	2.80	3.13	< 0.01	0.01	< 0.01	0.03
TI	3.26	3.57	< 0.01	< 0.01	< 0.01	0.02

De novo FA (C4:0 to C15:0); Mixed FA (C16:0 and C16:1); Preformed FA (> C16:1) *SFA* saturated fatty acids, *MUFA* monounsaturated fatty acids, *PUFA* polyunsaturated fatty acids, *CLA* conjugated linoleic acids, *LA* linoleic acid (C18:2n6), *ALA* alpha linolenic acid (C18:3n3), *AA* arachidonic acid (C20:4), *EPA* eicosapentaenoic acid (C20:5n3), *AI* atherogenicity index, *TI* thrombogenicity index, *SEM* standard error of the means

**Fig. 2:**
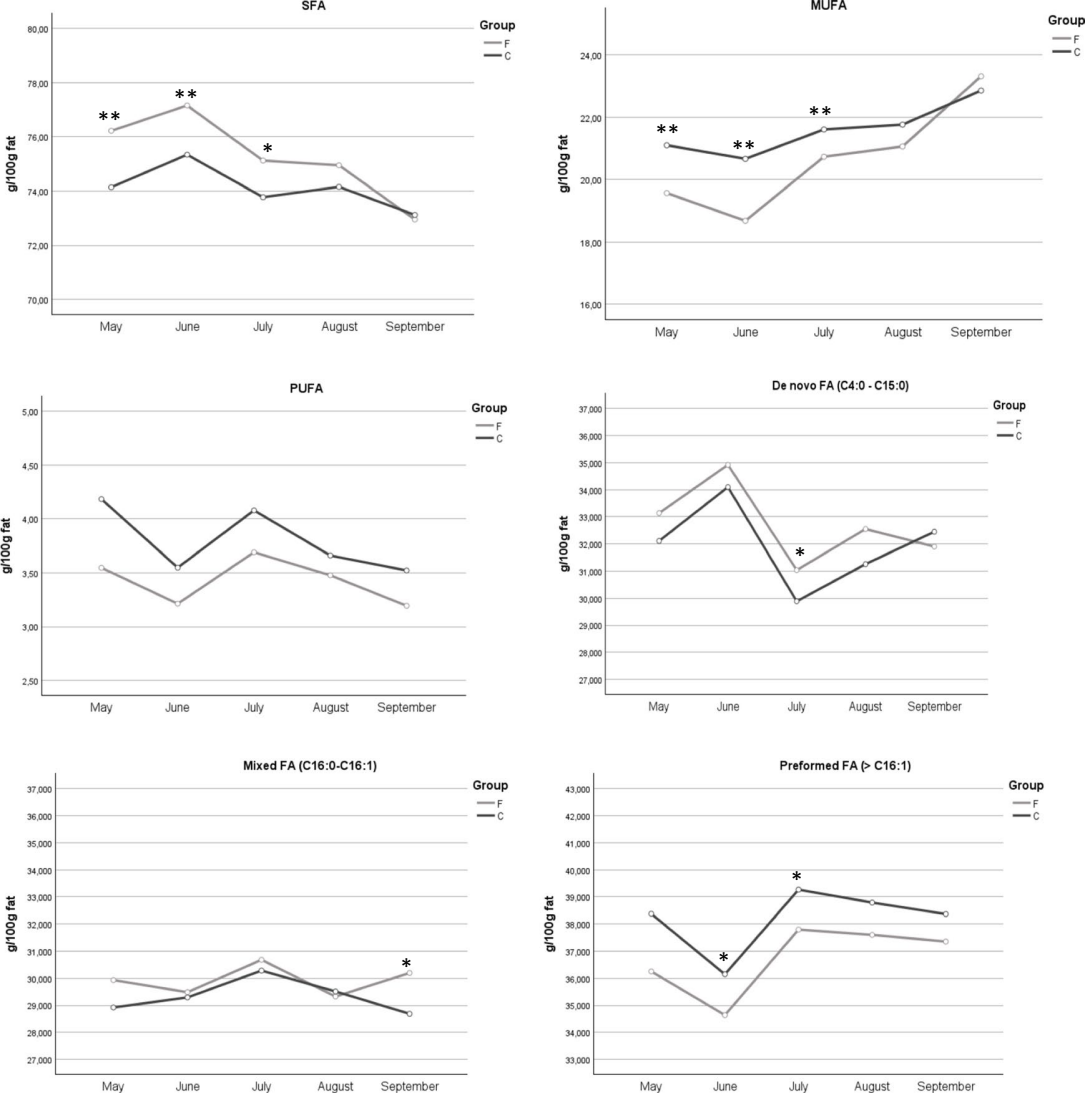
Main milk FA classes content during the trial in the experimental groups. FA: saturated fatty acids; MUFA: monounsaturated fatty acids; PUFA: polyunsaturated fatty acids; De novo FA (C4:0 to C15:0); Mixed FA (C16:0 and C16:1); Preformed FA (> C16:1). * = *P* < 0.05; ** = *P* < 0.01

Group F had lower content of all C18:1 isomers, except C18:1c11, also of C18:2 isomers, as CLA (C18:2c9 t11, C18:2 t10c12). The majority of FA and FA classes were affected by the sampling period (Fig. 2) and only some of them had interaction effects.

Milk from group F was characterized by a higher content in SFA (*P* < 0.01) and de novo FA whereas group C had higher content of MUFA, PUFA, n-3 and n-6 FA (Table 5). For all the classes of fatty acid a significant effect of the sampling period was observed, except for mixed FA (C16:0 and C16:1); the interaction between treatment and sampling period was significant for MUFA, SFA and n-6. AI and TI resulted both significantly lower (*P* < 0.01) in group C.

### Cheese chemical composition

Cheese composition and VOCs profile are reported in Tables 6 and 7. The cheese of group F had higher yield, moisture and lipid content but lower protein than control group.

**Table 6:** Cheese yield (g cheese/L milk) and composition expressed as g/100 g cheese

Item	Group C	Group F	G	P	GxP	SEM
			*P-value*			
Cheese yield	124	141	< 0.01	0.883	0.469	0.27
Protein	9.565	7.821	< 0.01	< 0.01	< 0.01	0.67
Fat	14.953	16.986	< 0.01	< 0.01	0.014	0.42
Moisture	64.685	66.226	< 0.01	< 0.01	0.240	0.89

*SEM* standard error of the means

**Table 7:** Volatile organic compounds (VOCs^*^) and total antioxidant capacity (TAC) of cheese

Item	Group C	Group F	Group effect	Period effect	GxP	SEM
			*P-value*			
Butanoic acid C4:0	3.355	3.210	0.664	0.289	0.314	0.16
Hexanoic acid C6:0	4.375	4.235	0.675	0.393	0.513	0.15
Nonanoic acid C9:0	4.700	3.780	0.021	0.880	0.459	0.20
Decanoic acid C10:0	6.165	5.130	0.012	0.417	0.451	0.22
Dodecanoic acid C12:0	16.475	14.205	< 0.01	0.094	0.082	0.39
Tetradecanoic acid C14:0	5.855	5.640	0.522	0.541	0.401	0.15
2.3-Butanediol	7.235	7.355	0.719	0.632	0.880	0.14
1-butanol-3-methyl	6.985	7.190	0.541	0.032	0.417	0.19
Phenylethyl alcohol	6.630	6.270	0.295	0.378	0.477	0.16
Ethyl hexanoate	5.030	4.875	0.643	0.798	0.868	0.14
Ethyl octanoate	9.455	6.200	< 0.01	0.003	0.003	0.58
Ethyl decanoate	6.915	7.875	0.017	0.017	0.010	0.29
2-Heptanone	5.655	5.470	0.581	0.128	0.117	0.18
2-Nonanone	12.835	15.220	< 0.01	0.356	0.030	0.41
Limonene	nd	3.350	< 0.01	0.023	0.223	0.53
Fenchone	nd	3.170	< 0.01	0.865	0.865	0.49
Estragole	nd	2.250	< 0.01	0.185	0.185	0.36
trans-Anethole	nd	2.600	< 0.01	0.766	0.766	0.40
TAC, mol FeSO_4_ equivalents/kg	2.070	3.410	< 0.01	0.363	0.532	0.25

^*^The values of volatile compounds are expressed as percent of total volatile compounds detected. SEM: standard error of the means

The major odour compounds detected in both cheeses were dodecanoic acid and 2-Nonanone.

Limonene, fenchone, estragole and trans-anethole, not usually detected in goat cheese, were highly different between the two groups, not being detected at all in group C. These are indeed the characteristic compounds of fennel seed. Among free fatty acids (FFA) nonanoic, decanoic and dodecanoic acids were higher in group C.

Cheese of fennel group had higher TAC than group C (*P* < 0.01). Indeed, the compounds characterizing fennel cheese, namely limonene, fenchone, estragole and trans-anethole are known for their strong antioxidant activity (Rather et al. 2016).

Cheese fatty acid profile is reported in Table 8. Among the de novo synthesized FA only C14:1 was significantly different between groups, being higher in group C (*P* < 0.05). With regard to C18:1 isomers, almost all of them were significantly different according to the treatment, to the period of sampling and to their interaction. This is the case of C18:1c6 which was higher in group C while C18:1c9, C18:1c10 and C18:1c12 were higher in group F. Linoleic acid (C18:2n6), gamma-linolenic (C18:3n6), alpha-linolenic and CLA isomers were higher in group F.

**Table 8:** Cheese fatty acid profile expressed as g/100 g of cheese fat

Item	Group C	Group F	Group effect	Period effect	G x P	SEM
			*P-value*			
C4:0	3.203	2.939	0.435	0.994	0.136	0.16
C6:0	2.137	1.705	0.217	0.125	0.202	0.19
C8:0	2.470	2.365	0.751	0.527	0.950	0.14
C10:0	9.734	10.278	0.129	0.568	0.637	0.16
C11:0	0.338	0.209	0.333	0.381	0.586	0.08
C12:0	3.628	3.589	0.906	0.948	0.542	0.14
C14:0	9.556	9.527	0.930	0.25	0.482	0.19
C14:1	0.345	0.219	0.026	0.969	0.793	0.03
C16:0	28.962	29.346	0.266	0.665	0.498	0.15
C16:1	0.351	0.308	< 0.01	0.211	0.010	0.01
C18:0	12.045	11.837	0.537	< 0.01	0.269	0.30
C18:1c6	0.102	0.027	< 0.01	< 0.01	< 0.01	0.02
C18:1n9	18.555	19.541	< 0.01	< 0.01	< 0.01	0.03
C18:1 t9	1.409	1.356	0.342	0.001	0.035	0.07
C18:1 t11	0.508	0.410	0.015	0.001	0.011	0.36
C18:1c10	0.251	0.312	< 0.01	< 0.01	< 0.01	0.03
C18:1c11	0.167	0.174	0.78	< 0.01	< 0.01	0.01
C18:1n12	0.092	0.107	< 0.01	0.79	< 0.01	0.01
C18:2 t6	0.244	0.321	< 0.01	< 0.01	< 0.01	0.02
C18:2 n6	1.327	1.456	0.013	0.174	0.864	0.04
C18:2c9 t11	0.409	0.505	< 0.01	< 0.01	0.087	0.02
C18:2 t10c12	0.197	0.222	0.003	0.025	0.428	0.01
C18:3n6	0.045	0.067	< 0.01	< 0.01	< 0.01	0.00
C18:3n3	0.838	0.918	< 0.01	< 0.01	0.223	0.02
C22:0	0.153	0.138	0.035	< 0.01	0.039	0.01
C20:3n6	0.012	0.021	< 0.01	< 0.01	< 0.01	0.00
C20:4	0.144	0.141	0.26	< 0.01	< 0.01	0.00
C22:2n6	0.013	0.011	< 0.01	< 0.01	< 0.01	0.00
C22:6n3	0.024	0.034	< 0.01	< 0.01	< 0.01	0.00
C24:1	0.026	0.020	< 0.01	< 0.01	< 0.01	0.00
SFA	74.209	73.544	< 0.01	< 0.01	< 0.01	0.243
MUFA	22.205	22.576	< 0.01	< 0.01	< 0.01	0.291
PUFA	2.925	3.238	< 0.01	< 0.01	0.008	0.082
n-3	0.949	1.035	< 0.01	< 0.01	0.657	0.023
n-6	2.395	2.543	< 0.01	< 0.01	< 0.01	0.044
n-6/n-3	2.521	2.462	< 0.01	< 0.01	< 0.01	0.038
Total CLA	0.606	0.727	< 0.01	< 0.01	0.116	0.028

*SFA* saturated fatty acids, *MUFA* monounsaturated fatty acids, *PUFA* polyunsaturated fatty acids, *CLA* conjugated linoleic acids, *SEM* standard error of the means

## Discussion

### Diet and pasture

Fennel seeds resulted rich in lipids, as also previously reported by Unal et al. (2013), representing an important source of bioactive compounds enhancing their therapeutic properties (Díaz-Maroto et al. 2006).

Fennel fatty acid profile was characterized by high MUFA content, mainly represented by petroselinic acid and low content of PUFA and SFA. Petroselinic acid is characteristic of the plant family *Apiaceae* and is particularly abundant in fennel oil according to Najdoska-Bogdanov et al. (2015). This compound has antimicrobial properties and is involved in the synthesis of lauric acid after its oxidation. Another peculiarity of fennel FA profile is the presence of C11:0 which, as monoacylglycerol preparation has an antimicrobial activity, especially against bacteria responsible for alimentary diseases (Doležalová et al. 2010).

Concerning pasture chemical composition, in the present trial in May the pasture had the lowest and the highest values of protein and NDF respectively, in contrast with our previous research in the same area (Lo Presti et al. 2023). This difference could be due to the high temperatures in spring 2022, which affected plant leaves apparatus, lowering the protein content. The increase of protein in July could be due to an increase in rainfall with a recovery of leaves.

The trend of FA in pasture was characterized by lower values of PUFA and higher of MUFA during June, July and August. This changes in PUFA/MUFA ratio could be due to climatic alterations that highly affect pasture productivity and nutritional composition. According to Martins-Noguerol et al. (2023) warming is able to increase plant productivity and at the same time it shifts the fatty acid profile, probably due to an alteration in plant community composition.

Both climatic stressors, warming and drought, might impact several plant physiology processes related to protein stability, membrane fluidity, changes in nitrogen metabolism, oxidative stress and photosynthetic activity.

### Milk

The group of goats supplemented with fennel seeds had a higher MY than the control group throughout the trial, as well as higher yields of fat, protein, and lactose. Similar results were reported by other authors (Moosavi-Zadeh et al. 2023; El-Hendawy et al. 2018; Fahim et al. 2022) for MY on cows, buffaloes and goats fed with *Foeniculum vulgare*. Nevertheless, El-Hendawy et al. (2018) and Fahim et al. (2022) recorded also increases in milk solid content contrary to Moosavi-Zadeh et al. (2023) and to our findings.

The increase in MY confirms the role of *Foeniculum vulgare* as a galactagogue in dairy goats as already demonstrated in women, even though its mechanism of action could be differently explained in ruminants. Indeed, the chemical similarity between trans-anethole, which is abundant in fennel, and dopamine makes it a suitable antagonist for dopamine receptors thus reducing its activity and enhancing PRL action on mammary gland. Since the role of PRL in ruminants’ lactation is controversial (Akers et al. 1981) other mechanisms of action could also be taken into consideration, which are mainly related to glucose metabolism.

Indeed, fennel seeds have orexigenic properties, being able to stimulate the appetite thanks to their aromatic compounds, mainly trans-anethole, estragole, and limonene, as showed in the work of Kargar et al. (2021) on calves and by Moosavi-Zadeh et al. (2023) on dairy cows. Moreover, when diet with same forage to concentrate ratio were fed to ruminants, fennel supplementation induced a reduction in the content of rumen acetate and increased the relative concentration of propionate, that is related to glucose synthesis (Moosavi-Zadeh et al. 2023).

The hyperglycaemic effect of fennel depends also on its oestrogenic properties, being able to affect insulin and glucose metabolism through the suppression of adipose lipolysis which reduces circulating FFA levels enhancing insulin sensitivity (Kim et al. 2014). Moreover, thanks to the presence of compounds that act as a dopamine antagonists, fennel increases glucocorticoid levels which are involved also in lactogenesis and galactopoiesis (Ahmadzadeh et al. 2006). Finally, another possible explanation for MY increase could be a regulatory effect of fennel compounds on the expression of *IGF1* (Insulin-Like Growth Factor 1) gene, as reported by Shahsavari et al. (2022). In their study the supplementation of mice diet with fennel seeds increased the expression of *IGF1*, a gene involved in the metabolism of glucose and in milk synthesis (Vernon 1989).

The changes in milk FA between the groups are probably due to the metabolic effect exerted by fennel compounds. Indeed, the lower content of preformed FA in group F and the higher content of the de novo FA suggests a lower uptake of body lipid reserve as observed also by Moosavi-Zadeh et al. (2023) on cows and may be explained by fennel properties to increase appetite and consequently intake of pasture or to improve diet digestibility. Moreover, as reported above, fennel seeds have hypolipidemic effects and stimulate insulin secretion through various mechanisms (Samadi-Noshahr et al. 2021; Kim et al. 2014) preventing body fat mobilization mainly by influencing the expression of insulin and leptin receptors genes (Zakernezhad et al. 2021).

Petroselinic acid was found to be present at a higher concentration in the milk of group C, albeit with a negligible difference. Consequently, while not detected, it is hypothesised that this FA was present in minimal amounts in the pasture, given the detection of a small amount of Apiacee in the pasture of the same area (Lopresti et al. 2023). This particular FA has been linked to the rumen metabolism of α-linolenic acid, as well as mammary desaturation of the resulting isomers (Martinez Marín et al. 2015). Additionally, the goat rumen microbiota has been observed to biohydrogenate unsaturated fatty acids, which suggests a potential modification of dietary petroselinic acid prior to its incorporation into milk fat (Martinez Marín et al. 2015). However, the extent to which this modification occurs and its effect on the levels of petroselinic acid in goat’s milk require further research. Among long chain FA, arachidonic acid was lower in group F. This result may be attributed to the high presence of petroselinic acid in fennel seeds, which has an inhibitory effect on the conversion of linoleic acid to arachidonic acid (Heidarian Miri et al. 2013).

As also observed by Moosavi-Zadeh et al. (2023) the content of C18:0 and C18:1n9 did not change between groups. According to Gross et al. (2011) these long-chain FAs in milk could be used as indicators for evaluating energy status in dairy cows and as early predictor of negative energy balance, which suggests that in our trial all goats’ energy requirements were satisfied even in group F, having higher milk production.

The range values obtained for milk AI and TI fall within the results reported for goats’ milk in the literature (Osmari et al. 2011). Furthermore, despite the observed variation between the groups, according to Silanikove et al. (2010), these values validate the superior quality of milk obtained from grazing goats in comparison to those from goats fed indoors. The effect of sampling period appeared to be particularly significant for the majority of FA classes. As reported also by other authors (Strzałkowska et al. 2009; Tudisco et al. 2010; Bernard et al. 2021) the stage of lactation highly affects milk FA composition. Indeed, the sampling effects were observed for the de novo synthesized and the preformed FA, the latter due to the lipomobilization that generally occurs in a more intensive way during the first stages of lactation (Chilliard et al. 2006).

With regards to FA classes, group F had lower content of MUFA and PUFA and higher of SFA. Similar results were observed by Moosavi-Zadeh et al. (2023) on cows in early lactation except for SFA. On the opposite, Mahmoud et al. (2020) found lower SFA and higher MUFA in milk from cows in mid lactation supplemented with fennel.

SFA, PUFA and MUFA showed treatment, sampling and interaction effects. It is well known that in goats’ milk SFA tend to decrease during the stage of lactation while MUFA have an opposite trend. In the present study, the experimental groups exhibited a comparable trend, with group F registering lower content of MUFA and higher of SFA during almost the entire lactation stages.

### Cheese

Feeding of fennel seeds in goat diet resulted in higher cheese yield and fat content compared to control group. Cheese yield is affected by many factors (milk composition, genetic variants, pasteurization, coagulant type etc.) but among them, protein and fat content are considered the most influent (Abd El- Gawad and Ahmed 2011). As observed by Guinee et al. (2007) the increase of milk protein content during the lactation leads to an increase in cheese yield that has a similar trend to the protein content.

The increased cheese yield may be attributable to the elevated moisture content, which is contingent on numerous factors. A primary factor identified as contributing to this variation is the animals’ diet, with changes in moisture content during the season being observed due to variations in the quality of pasture (Kefford et al. 1995) and due to different calcium levels, which directly decrease moisture (McMahon et al. 2005). Furthermore, the composition of FA in cheese has been demonstrated to influence its matrix and moisture-holding capacity (McMahon et al. 2005). As El-Metwally et al. (2023) have reported, an increase in moisture content has been observed in cheese when it is richer in unsaturated fatty acids, a phenomenon that may also have been observed in the present study.

The changes in cheese fat and protein content could be explained by the different microflora developed on F group cheese that may have led to more proteolysis than lipolysis. In the work of Ismail et al. (2004) no cheese yield increase was found when goats were supplemented with fennel. In addition, in their work the authors found a reduction of lipolytic bacteria and a decrease of coliformis counts compared to control group.

With regard to VOCs composition, in dairy products it can be found a great variety of class of compounds, such as, alcohols, ketones, aldehydes, ester, FFA, terpenes that could change according to different feeding strategies (Balivo et al. 2023). The fat appears to be the main factor affecting flavour formation (Vagenas and Roussis 2012) due to the lipolysis occurring during the ripening which yields FA. In cheeses in general, the activity of lipolysis, mainly exerted by endogenous and microbial lipases, led to a greater formation of short chain fatty acids (SCFA) which play a more significant role in the flavour of dairy products than medium and long chain FAs. A proportion of FFA, especially of short chain, also derived from the lactose and protein degradation.

In the present study, the major odour compounds detected in both cheeses were dodecanoic acid and 2-nonanone which are generally identified in goat cheese in different quantities.

The methyl ketone 2-nonanone, generally associated with fruity flavour (Chiofalo et al. 2004), resulted higher in group fed fennel whereas decanoic and dodecanoic acids were higher in group control. These two FA have been recognized as the main responsible for goat flavour in dairy products (Singh et al. 2003). They generally derived from the lipolysis that occurs in cheese during the ripening and represent indicators of the process of lipolysis in milk. The use of antioxidants in cheese industry is generally aimed to reduce the phenomena of lipid oxidation over the ripening period. Fennel seeds indeed, have been tested in cheese production for reducing the lipolytic trend in the work of Mansha Rafiq and Ghosh (2021). In their work the authors demonstrated that by adding fennel extract at a level of 1.2% of total solids at the end of cheese processing, the organoleptic properties and rate of lipolysis and proteolysis decreased. In our trial the higher yield, justified also by a higher moisture content (*P* < 0.05), may have created a better condition for proteolytic bacteria proliferation, leading to a reduction of protein content and an increase of protein derived products.

Fennel seeds supplementation highly affected cheese VOCs profile, providing 4 specific aromatic compounds, namely limonene, fenchone, estragole and trans-Anethole which are usually not detected in goats cheese and that are the main responsible of fennel well known antioxidant properties (Rather et al. 2016). In particular, limonene has been associated to fresh fruity flavour, fenchone to camphor flavour and trans-anethole to anise flavour. In the work of Ismail et al. (2004), authors used a group of panellists to rate the likeability of cheese, with positive results for cheese obtained from goats supplemented with fennel seeds.

The FA profile of cheese also resulted affected by fennel supplementation, revealing several differences between the groups. The cheese from the fennel group had higher levels of MUFA, PUFA, n-3, n-6, and total CLA content compared to group C. With regard to the other main differences, the cheese of group F had higher content of C18:1c10, petroselinic acid, linoleic acid and alpha-linolenic acid and CLA isomers, the last known as beneficial for human health (Trinchese et al. 2019). During cheese ripening, as for others processed and stored foods, oxidation of FA occurs, more intensively on PUFA (Tao 2015). The results obtained on cheese may suggest that thanks to its antioxidant properties, fennel seeds, specifically their compounds which had been transferred in cheese (trans-anethole, limonene, fenchone and estragole), were able to protect long chain and unsaturated FA from oxidation, revealing significant differences from control group.

## Conclusion

The findings of our study suggest that 15 g/head/day of fennel seeds are able to increase milk in grazing goats, confirming the galactagogue properties of *Foeniculum vulgare*. Significant increase in cheese yield has also be observed although this difference can be explained by the difference in moisture. The fatty acid profile of milk was affected by the supplementation especially for de novo FA which were higher in fennel fed goats whereas the milk of control group showed higher PUFA and MUFA contents. Four different aromatic compounds were detected exclusively in cheese from goat fed fennel which was indeed characterized also by a higher antioxidant activity and higher MUFA, omega 3, omega 6 and total CLA content. Therefore, these observations confirm the antioxidant properties of fennel, traditionally used as natural preservatives in food industry. Further studies could be aimed at evaluating the effect of different levels of inclusion and longer ripening times when assessing cheeses.

## Data Availability

The datasets generated during and/or analysed during the current study are available from the corresponding author on reasonable request.
